# 

**Published:** 1788

**Authors:** 


### CATALOGUE OF BOOKS.

1. CONSIDERATIONS on Bilious Difeafes; and fome particular Affections of the Liver and the Gall Bladder. By *John Andree*, M. D. 8vo. Hertford, 1788.

2. An Effay on the Caufes of the Variety of Complexion and Figure in the Human Species. To which are added, Strictures on Lord Kaimes's Difcourfe on the original Diverfity of Mankind. By the Rev. *Samuel Stanhope Smith*, D. D. Vice Prefident

President and Profeffor of Moral Philofophy in the College of New Jerfey, and Member of the American Philofophical Society (held at Philadelphia) for promoting ufeful Knowledge. A new Edition, with Additions, by way of Notes, by a Gentleman of the Univerfity of Edinburgh. 8vo. Edinburgh, 1788.

3. Bath Waters; a conjectural Idea of their Nature and Qualities, in three Letters to ——. To which are added, Putridity and Infection unjuftly imputed to Fevers, a cruel public Grievance, attempted to be redreffed; with fome Account of the Nature and Management of plain Fevers. By *A. W.* M. D. Reg. Coll. Med. Edinb. Soc. 8vo. *Robinfons*, London, 1788.

4. Letters to Sir Jofeph Banks, Baronet, Prefident of the Royal Society, on the Subject of Cochineal Infects difcovered at Madras. By *James Anderfon*, M. D.: with a Copper-plate Engraving annexed of the different Infects mentioned in the letters, from the drawings of Baron Reichel. Alfo an Engraving of the Opuntia major, fpinulis obtufis, mollibus & innocentibus; and the Plan of a Nopalry in the Bifhopric of Guaxaca, in the Kingdom of Mexico, extracted from the fecond Volume of

Sir

Sir Hans Sloane's Hiſtory of Jamaica, for the Uſe of Gentlemen in India, who may be diſpoſed to make plantations, and are not in poſſeſſion of that Work. 8vo. Madras, 1788.

5. Critical Introduction to the Study of Fevers. Read at the College of Phyſicians for the Gulſtonian Lectures; by *Francis Riollay*, M. D. Fellow of the College. 8vo. *Cadell*, London, 1788.

6. An Eſſay on the epidemic Diſeaſe of Lying-in Women of the Years 1787 and 1788. By *John Clarke*, Licentiate in Midwifery of the Royal College of Phyſicians, and Teacher of Midwifery in London. 4to. *Johnſon*, London, 1788.

7. The Elements of Medicine, tranſlated from the Elementa Medicinæ *Brunonis*, with large Notes, Illuſtrations, and Comments, by *John Brown*, M. D. Author of the original Work. 8vo. 2 Vols. *Johnſon*, London, 1788.

8. Obſervations on the Medical Practice of Dr. *Brown*; or, An Inquiry into the Abuſe of Stimulants in Fevers. 8vo. *Gardner*, London, 1788.

9. The Medical Reform; containing a Plan for the Eſtabliſhment of a Medical Court of

Judicature,

Judicature, to correct Abuses of the Profession of Physic in all its Branches; and a Medical College, to give full Instruction to Youth intended as Surgeons for the Navy or Army, without Expence to the Nation, or Oppression to Individuals: Being a Letter to the Right Honourable William Pitt, Chancellor of the Exchequer, &c. 8vo. *Deighton*, London, 1788.

10. Practical Dissertations on Nervous Complaints, and other Diseases incident to the Human Body: With an historical Investigation of their Causes and Cure; in which are interspersed some singular Cases. By Mr. *Neale*, late Surgeon of His Majesty's fifth Regiment of Infantry, &c. 8vo. *Faulder*, London, 1788.

11. First Lines of the Theory and Practice of Philosophical Chemistry. By *John Berkenhout*, M. D. 8vo. *Cadell*, London, 1788.

12. An Essay on the Nature and Origin of the Contagion of Fevers. By *John Alderson*, M. D. Member of the Royal Medical Society of Edinburgh. 8vo. *Murray*, London, 1788.

13. Thoughts on the Cancer of the Breast. By *George Bell*, Surgeon at Redditch. 8vo. *Johnson*, London, 1788.

14. Floræ

14. Floræ Cantabrigiensi Supplementum alterum *. Auctore *Richardo Relhan*, A. M. Collegii Regalis Capellano, Regiæ Societatis Londinensis Socio. 8vo. Cantabrigiæ, 1788.

15. Flora Caroliniana, secundum Systema Vegetabilium perillustris Linnæi digesta; Characteres essentiales naturalesve & differentias veras exhibens; cum emendationibus numerosis descriptionum antea evulgatarum: adumbrationes stirpium plus mille continens: nec non, generibus novis non paucis, speciebus plurimis novisq. ornata. Auctore *Thomas Walter*, Agricola. 8vo. Londini, 1788.

16. Anatomicarum Annotationum liber secundus † de Organo Olfactus præcipuo deque nervis nasalibus interioribus e pari quinto nervorum Cerebri. Auctore *Antonio Scarpa*, in Ticinensi Archigymnasio Anatomes & Chirurgicarum operationum professore, R. Scientiarum Acad. Berolinensis, Cæsareo-Leopold. Nat. Curios. R. Medic. Parisiens. Societ. &c. Sodali. 4to. Ticini Regii, 1785. c. Tab. æneis.

17. Dissertatio Botanica de Moræa, quam

* See Vol. VI. page 333, and Vol. VII. page 435.

† See Vol. I. page 212.

præside *Carol. Pet. Thunberg*, Equite aurat. Reg. ord. de Vasa, Med. Doct. Prof. Med. & Botan. reg. & ord. &c. publice Examinandam sistit *Zacharias Colliander*, Stipend. Reg. Smolandus. 4to. Upsaliæ, 1787.

18. De Usu Inustionum vario & præcipue in Gangræna metastatica exoptato, præside *Adolpho Murray*, pro gradu Doctoris rite impetrando disserit *Joh. Frederic. Sachlen*. 4to. Upsaliæ, 1787.

19. Dissertatio Inauguralis medica Observationes * quasdam medicas continens. Auctore *Joanne Friderico Schwartze*, Dipholza-Hanoverano. 4to. Gottingæ, 1787.

20. Pelvis Animantium Brutorum cum Humana comparatio. Specimen primum. Scripsit *Bernhardus Gottlob Schregerus*, M. B. 4to. Lipsiæ, 1787.

21. De usu Cinnamomi in partu valde dubio. Auctore *Joanne Carolo Gehler*, Pathol. Prof. p. o. &c. 4to. Lipsiæ, 1787.

22. *Caroli Ludovici Willdenow* Societ. Natur. Curios. Halens. Sodal. Floræ Berolinensis Prodromus secundum Systema Linneanum ab illust.

* De inflammatione Hepatis. — De morbis venereis larvatis.

Viro

Viro ac Eq. C. P. Thunbergio emendatum conſcriptus: cum Tabulis VII. æri inciſis. 8vo. Berolini, 1787.

23. Diſpenſatorium Fuldenſe tripartitum tam patriæ uſibus, quam ſæculi moderni (noſtri) genio accomodatum a *Franc. Ant. Schlereth*, Phil. & Med. D. Conſiliario intimo & archiatro, &c. 8vo. Fulda, 1787.

24. Tentamen Phyſico-medicum de Electricitate. Auctore *Gulielmo Allanby*, Anglo-Britanno. 8vo. Edin. 1788.

25. Diſſertatio Medica Inauguralis, quædam de Dentitione Morbiſque ex ea pendentibus complectens. Auctore *Samuel Allvey*, Anglo. 8vo. Edin. 1788.

26. Diſſertatio Inauguralis quædam de effectibus pathematum exhibens. Auctore *Carolo Joanne Berkley*, Anglo. 8vo. Edin. 1788.

27. Tentamen Phyſiologico-medicum de uſu & effectu aeris puri in corpus humanum. Auctore *Henrico Burton*, Anglo-Britanno. 8vo. Edin. 1788.

28. Diſſertatio Medica Inauguralis de Phthiſi pulmonali ſcrophuloſa. Auctore *Thoma Concanen*, Hiberno. 8vo. Edin. 1788.

29. Tentamen Medicum Inaugurale de Vitiis quibus Humores corrumpi dicuntur, eorumque remediis. Auctore *Samuele Crumpe*, Hiberno. 8vo. Edin. 1788.

30. Tentamen Physico-medicum Inaugurale quædam de Strabismo complectens. Auctore *Roberto Graves*, Anglo-Britanno. 8vo. Edin. 1788.

31. Tentamen Inaugurale de Phthisi pulmonali scrofulosa. Auctore *Gulielmo Saunders O'Halloran*, Hiberno. 8vo. Edin. 1788.

32. Tentamen Physiologicum de Nutrimine Fœtus Humani. Auctore *Gulielmo Handy*, de Urbe apud Rhodam Insulam Newport dicta. 8vo. Edin. 1788.

33. Dissertatio Inauguralis de Actione & Usu Emeticorum. Auctore *Jacobo Moultrie*, Carolinensi Australi. 8vo. Edin. 1788.

34. Dissertatio Medica Inauguralis de Ictero. Auctore *Gulielmo Quillin*, ex Insula Mona. 8vo. Edin. 1788.

35. Dissertatio Medica Inauguralis de Fœtus Humani Nutrimento. Auctore *Jacobo Robertson*, Scoto-Britanno. 8vo. Edin. 1788.

36. Tentamen Medicum Inaugurale de Testium Tumore Gonorrhœæ superveniente. Auctore *Jacobo Short*, Scoto-Britanno. 8vo. Edin. 1788.

37. Dis-

37. Dissertatio Medica Inauguralis de Inflammatione pneumonica. Auctore *Francisco Smith*, Anglo. 8vo. Edin. 1788.

38. Dissertatio Medica Inauguralis de Amenorrhœa. Auctore *Jacobo Watson*, Scoto. 8vo. Edin. 1788.

39. Dissertatio Medica Inauguralis de Tetano. Auctore *Josepho Nicholes Wilson*, Carolinensi Meridionali. 8vo. Edin. 1788.

40. Precis du Siecle de Paracelse, par M. *Joyand*, D. M. de la faculté de Besançon, Médecin de l'Hopital militaire de Brest. Tome 1. 8vo. Paris, 1788.

41. Methode pour traiter toutes les Maladies, très utile aux jeunes Médecins, aux Chirurgiens, et aux gens charitables qui exercent la Medecine dans les campagnes; dediée au Roi; par M. *Vachier*, Docteur Regent de la Faculté de Medecine, ancien Professeur des Ecoles de Medecine de Paris, Docteur en Medecine de l'Université de Montpellier. Tomes VII. 12mo. Paris, 1786-8.

42. Demonstrations elementaires de Botanique, contenant les Principes generaux de cette Science, l'Explication des termes, les Fondemens des Methodes, et les Elemens de la Physique des Vegetaux; la Description des Plantes les plus comcommunes, les plus curieuſes, les plus utiles, rangées ſuivant la Methode de M. *de Tournefort*, et celle du Chevalier *Liné*; leurs Uſages et leurs Proprietés dans les Arts, l'Economie rurale, dans la Medecine humaine et veterinaire, ainſi qu'une Inſtruction ſur la formation d'un Herbier, ſur la deſſiccation, la maceration, l'infuſion des plantes, &c. Troiſième edition, corrigée, et conſiderablement augmentée. Tomes 3. 8vo. Lyon, 1787.

43. Traité des principales et des plus frequentes Maladies externes et internes, à l'uſage des jeunes Docteurs en Medecine, des Chirurgiens-medecins, et de Praticiens qui ſuppleent au defaut des Medecins gradués, ainſi qu'a celui des perſonnes eclairées, qui, par des motifs de bienfaiſance, exercent la Medecine dans les Campagnes; dedié a L. L. E. E. les Souverains Seigneurs de l'Etat de Berne; par M. *Jean Freder. de Herrenſchwand*, Docteur en Medecine, Aſſocié etranger de la Société Royale de Medecine de Paris, et de la Société economique de Berne, ci-devant premier Medecin du Roi de Pologne, et Conſeiller intime de S. M. et de la ſereniſſime Cour de Saxe-Gotha, Medecin conſultant de la Ville de Berne. 4to. Berne, 1788.

44. Obſervations generales ſur les Hopitaux,

ſuivies

ſuivies d'un projét d'Hopital; par M. *Iberti*, Docteur en Medecine, avec des plans detaillés, redigés, et deſſinés par M. *Delannoy*, Architecte, et ancien penſionnaire du Roi a Rome. 8vo. Paris, 1788.

45. Traité des Hernies, de M. *Aug. Gottlieb Richter*, Medecin et Conſeiller de la Cour de S. M. Britannique, Profeſſeur de Medecine et de Chirurgie en l'Univerſité, Preſident du College des Chirurgiens, Directeur de l'Hopital academique de Gottingue, Membre de l'Academie Royale des Sciences de cette Ville, de celle de Stockholm, et de la Société de Medecine de Copenhague; traduit de l'Allemand ſur la ſeconde Edition, par *Joſeph Claude Rougemont*, Docteur en Medecine, Profeſſeur d'Anatomie et de Chirurgie en l'Univerſité Electorale de Bonn ſur le Rhin, et ancien Demonſtrateur d'Anatomie et de Chirurgie a l'Hopital de Breſt. 4to. Bonn, 1788.

46. Memoires couronnés en l'année 1786, par l'Academie des Sciences, Belles Lettres et Arts de Lyon, ſur l'Utilité des Lichens dans la Medecine et dans les Arts; par M. M. *Hoffmann*, D. M. *Amoreux*, fils, D. M. et *Willemet*, profeſſeur de Chimie et de Botanique. 8vo. Lyon, 1787.

47. Des Propriétés de la Plante appellée Rhusradicans; de son Utilité, et des Succés qu'on en a obtenus pour la guerison des Dartres, des Affections dartreuses, et de la Paralysie des parties inferieures, &c;. Par M. *du Fresnoy*, Docteur en Medecine de l'Université de Montpellier, Conseiller du Roi, Médecin des Camps et Armées de S. M. ancien Médecin de ses Armées en Allemagne, de la Société Royale des Sciences de Montpellier. 8vo. Paris, 1788.

An accidental circumstance, it seems, led to a trial of the rhus radicans in cutaneous complaints. A person who had rubbed the leaves of this plant between his fingers, complained of much heat and itching of his hands, which swelled and became covered with small vesicles filled with a yellowish fluid. This erysipelatous affection soon extended over every part of his body; but at the end of about ten days, when the inflammation and swelling had entirely subsided, he found himself cured of an herpetic blotch on one of his wrists, which, for the space of six years, had resisted a variety of mercurial and other remedies. After this our author tried it with success in nine cases of obstinate cutaneous eruption. In one of these he employed an infusion, and in the others a water distilled from the leaves of the plant.—Five instances

ſtances are mentioned of the good effects of theſe remedies in paralytic affections.

48. Recherches ſur l'endurciſſement du tiſſu cellulaire des enfans nouveaux-nés ; extraites des volumes de la Société Royale de medecine ; par M. *Andry*. 4to. Paris, 1788.

49. Analyſe chemique de l'eau ſulfureuſe d'Enghien, pour ſervir a l'hiſtoire des eaux ſulfureuſés en general, par M. M. de *Fourcroy*, Medecin de la Faculté de Paris, de l'Academie Royale des Sciences, &c. et *de la Porte*, Medecin de la Faculté de Paris, &c. 8vo. Paris, 1788.

50. Abhandlung ueber die veneriſche Krankheit; von *Chriſtoph Girtanner*, der Arzneywiſſenſchaft und Wundarzneykunſt Doctor, der koenigl. Societaet der Wiſſenchaften zu Goettingen Correſpondenten. 8vo. Goettingen, 1788.

This volume contains a valuable treatiſe on the venereal diſeaſe, from which we have extracted an account of the aſtragalus exſcapus*. It contains much curious matter, relative to the hiſtory of the diſeaſe, and in particular, ſome new and forcible arguments, deduced chiefly from the Spaniſh writers, in proof of its having been

* See page 405.

 brought brought from America to Europe. In another volume the author propofes to give a catalogue of all the writings on this difeafe, that have been publifhed frcm the time of its firft appearance to the prefent, with a fhort account of what is particularly worthy of notice in each. For this purpofe, it feems, he has looked over two thoufand different publications, of which, upwards of three hundred, he obferves, were unknown to Aftruc, tho' of a date prior to that of his work on the venereal difeafe, which appeared in the year 1740.

51. Verhaltungfregeln fuer Schwangere und Kindbetteringen, *i. e.* Directions for the management of pregnant and lying-in-women. By *Raphael Steidele*, public teacher of Surgery and Midwifery in the general hofpital (at Vienna). 8vo. Vienna, 1787.

52. Chemifcher Lehrbegriff nach Spielmanns grundfaetzen aufgearbeitet, und mit den neueften erfahrungen bereichert, *i. e.* A chemical text book, drawn up according to Spielmann's principles, and enriched with the lateft difcoveries. By *G. F. C. Fuchs*, M. D. Profeffor of Phyfic at Jena. 8vo. Leipfic, 1787.

53. Grundlinien oder Revifion der ganzen praktifchen Arzeneikunde; ein handbuch und leitfaden fuer praktische Aerzte und Wundaerzte, *i.e.* Principles, or Review of the whole practice of physic; intended as a compendium and guide for practical Physicians and Surgeons. 8vo. Leipsic, 1787.

54. Dr. *Th. Skeete* Erfahrungen und Beobachtungen ueber die rohrichte und rothe Peruvianische Rinde, nebst einer anleitung, die Fieber, die Braune und andere Krankheiten zu heilen. Aus dem Englischen. nebst einigen beylagen des Deutschen Herausgebers. 8vo. Leipsic, 1787.

55. Versuch ueber die einimpfung der Pocken, *i. e.* An essay on the inoculation of the small-pox. By *H. J. Raffler*, M. D. 8vo. Graetz, 1787.

56. Physikalische und Philosophische abhandlungen der Gesellschaft der Wissenschaften zu Manchester. Erster Theil, aus dem Englischen. 8vo. Leipsic, 1788.

57. Dissertatio Psychologica inauguralis de Sympathia. Auctore *Engelbert Schlickum*. 8vo. Duisburgh, 1788.

58. *J. Ph. Vogler*, Pharmaca selecta observationibus clinicis comprobata denuo edita et aucta. 8vo. Wetslar, 1788.

59. Memoires pour servir a l'Histoire Physique et Naturelle de la Suisse, rediges par M. *Reynier*,

Membre de plusieurs Sociétés, et par M. *Struve*, Professeur honoraire de Chymie á l'Academie de Lausanne, et Membre de plusieurs Sociétés. Tome 1er. 8vo. Lausanne, 1788.

60. Quinta Dissertatio Botanica de Sterculia, Kleinhovia, Ayenia, Buttneria, Bombace, Adansonia, Crinodendro, Aytonia, Malachodendro, Stewartia et Napæa. Accedit præcedentium Dissertationum Mantissa 36 Tabulis ære incisis ornata, Auctore *Antonio Josepho Cavanilles*, Hispano-Valentino. 4to. Parisiis, 1788.

61. Sexta Dissertatio Botanica de Camellia, Gordonia, Morisonia, Gossypio, Waltheria, Melochia, Mahernia, Hermannia, Urena, Halesia, Styrace, Galaxia, Ferraria, et Sisyrinchio. Accedit Mantissa tertia; 41 Tabulis ære incisis ornata, Auctore *Antonio Josepho Cavanilles*, Hispano-Valentino. 4to. Parisiis, 1788.

62. Dissertatio Medica Inauguralis de Caryophyllis aromaticis quam præside *C. P. Thunberg*, Eq. aur. Reg. ord. de Vasa, Med. Doct. Prof. Med. et Botan. Reg. et ord. &c. pro gradu publicé ventilandam offert *Herman. Rud. Hast*, Medic. Provinc. Ostroboth. 4to. Upsaliæ, 1788.

63. Restio, quem Dissertatione Botanica, præside *C. P. Thunberg*, Eq. aur. Reg. ord. de Vasa, &c. publico examini subjicit *Petrus Lundmark*, Nericius. 4to. Upsaliæ, 1788.

64. De

64. De Myriſtica, præſide *C. P. Thunberg*, Eq. aur. Reg. ord. de Vaſa, &c. pro gradu Doctoris differit *Fredericus Vilhelm. Radloff*, Stockholmienſis. 4to. Upſaliæ, 1788. c. tab. æn.

65. Arbor Toxicaria Macaſſarienſis, quam præſide *C. P. Thunberg*, Eq. aur. reg. ord. de Vaſa, &c. pro gradu Doctoris publico ſubjicit examini *Chriſten Æjmelæus*, Fenno. 4to. Upſaliæ, 1788.

66. De Moxæ atque Ignis in Medicina rationali uſu, ſub moderamine *C. P. Thunberg*, Eq. aur. &c. pro gradu Medico differit *Johannes Guſtavus Hallmann*, Reg. Coll. Med. Membr. et Medicus pauperum Holmiæ, Stockholmienſis. 4to. Upſaliæ, 1788.

67. De Veneni Animalium Rabidorum Natura, ejuſque Medela, Diſſertatio Inagauralis, Auctore *Friderico Anſelmo Brevel*, Schneebergenſi. 4to. Lipſiæ, 1788.

68. De Acido corporum Vegetabilium Elementari, ejuſque varia modificatione. Auctore *Solom. Conſtantino Titio*, Vitebergenſi. 4to. Lipſiæ, 1788.

69. Hiſtoria Tumoris et Hæmorrhagiæ Alveolaris Chronicæ, feliciter ſanatæ. Auctore *C. C. Siebold*, Phil. et Med. Doctore, &c. 4to. Wurtzburg, 1788.

70. *A. Guil. Roth,* Tentamen Floræ Germanicæ. Tom. I. continens enumerationem plantarum in Germania ſponte naſcentium. 8vo. Lipſiæ, 1788.

71. Obſervationes de Oeſtro Ovino atque Bovino factæ. Auctore *Johanne Leonhard. Fiſcher,* Franco-Baruthino; cum tabulis æneis quatuor. 4to. Lipſiæ, 1788.

72. *C. Guil. Scheele* Opuſcula Chemica & Phyſica latine vertit *G. H. Schaefer,* Vol. I. Edid. & Præfatus eſt *E. B. G. Hebenſtreit.* 8vo. Lipſiæ, 1788.

73. Enchiridion Hiſtoriæ naturali inſerviens, quo Termini & Delineationes ad Avium, Piſcium, Inſectorum & Plantarum adumbrationes intelligendas & concinnandas, ſecundum Methodum Syſtematis Linnæani continentur, Editore *Jo. Reinholdo Forſter,* LL. Med. & Philoſ. D. & LL. AA. M. 8vo. Halæ, 1788.

74. Spicilegium Obſervationum de Aconito. Auctore *J. L. Koelle,* cum ic. aer. inc. 8vo. Erlang. 1788.

75. Diſſertatio Inauguralis Medica de noxa & abuſu Clyſmatum. Auctore *Carolo Friderico Gotthelf Schaeffer,* Elterleina-Miſnico. 4to. Wittenbergæ, 1788.

75. Diſ-

76. Dissertatio Medica Inauguralis de Melancholia ex Mente. Auctore *Casparo Landis*, Tigurino-Helveto. 8vo. Goettingæ, 1788.

77. Dissertatio Medica Inauguralis de Malignitate Febrium. Auctore *Carolo Kries*, Thorunensi. 8vo. Goettingæ, 1788.

78. De morbis Gastricis Phthisin mentientibus, Dissertatio Inauguralis Medica. Auctore *Geo. Wolfgang Eichhorn*, Norimbergensi. 8vo. Goettingæ, 1788.

79. De Sale Ammoniaco; Dissertatio Inauguralis Medica. Auctore *Gerh. And. Rud. Schmid*, Hannoverano. 8vo. Goettingæ, 1788.

80. Dissertatio Inauguralis Medica sistens momenta quædam circa sexus differentiam. Auctore *Adolpho Friderico Nolde*, Neostrelitiensi Megapolitano. 8vo. Goettingæ, 1788.

81. *Petri Orlandi*, Romani, Philosophiæ ac Medicinæ Doctoris, de Variolarum refellenda Inoculatione Dissertatio. 8vo. Romæ, 1788.

INDEX.

### I N D E X.

A. Page

ACADEMY of Sciences, at Paris, Report relative to Hofpitals, — — 215
Academy, Medico-chirurgical, at Vienna, Tranfactions of, ——— ——— 100
Aeimelæus, C. de Arbore Toxicaria, — 431
Alderfon, Dr. J. on the Contagion of Fevers, — 418
Allanby, Gul. de Electricitate, ——— 421
Allvey, Samuel, de Dentitione, ——— *ibid.*
Amaurofis, Cafe of, cured by Electricity, — 389
Amputation, Obfervations relative to, — 117, 223
——— in the Middle of the Foot, Cafe of, 54
Anderfon, Dr. J. on Cochineal Infects, — 416
Andree, Dr. J. on bilious Difeafes, ——— 415
Andry, M. fur l'Endurciffement du Tiffu cellulaire, 427
Aneurifm, Obfervations on the fpontaneous Cure of, 142
Arnemann, J. ueber das Gehirn, — 100
Arfenic, Efficacy of, in Intermittents, — 48
Afh, Dr. J. on the Waters of Spa, — 326
Afhton, Petrus, de Febre puerperarum, — 91
Affalini, Sig. fui vafi Linfatici, ——— 217
Aftragalus Exfcapus, its Effects in the Lues Venerea, 405

B.

Bains de Geifmar, Defcription des, — 214
Bandages, elaftic, Remarks on, ——— 44
Bath Waters, Narrative of their Efficacy, - 86
———, conjectural Idea of their Nature, 416
Belcombe, Gul. circa Motum Bilis, ——— 102
Bell, Benjamin, Syftem of Surgery, ——— 208
———, George, on the Cancer of the Breaft, — 418
Benjamin Tree, botanical Defcription of, — 80
Beretta, F. della Febbre epid. dell' Anno 1783, 216
Bergman, Torb. Amminelfe-Tal ofver, — 99
———, de Syft. Foffilium nat. — 330
Berkenhout, Dr. J. Philofophical Chemiftry, - 418
Berkley, C. J. de affectibus Pathematum, — 421
Berrettini, P. Tabulæ Anatomicæ, — 327

Bew,

Page

Bew, Dr. Geo. of the epidemic Catarrh, — 354

Blackburne, Dr. W. on the Effects of a large Dose of Emetic Tartar, — — 61

Blumenbach, J. F. Index Scriptorum Med. Goettingensium, — — 212

Boelke, J. C. T. de Mercurio Tartarisato, — 102

Bolton, James, History of Fungusses, — 88

Bonomo, Sig. his Account of the Itch Insect, — 31

Botanique, Demonstrations elementaires de, — 423

Bouvart, M. Eloge historique de, — 96

Brand, Tho. Case of a Boy mistaken for a Girl, 86

Brevel, F. A. de veneno anim. rabidorum, — 431

Brown, Dr. John, Elements of Medicine, — 417

——————, Observations on, *ib.*

Burton, Henr. de Usu & Affectu Aeris puri, — 421

C.

Caldwall, Jac. de Nephritide, — 91

Cappel, J. F. ueber den Blattern, — 100

—————— die Englische krankheit, - 216

Carmichael, Joannes, de Fermentatione, — 91

Cataract, Extraction and Depression of, in the same Patient, — — 109

Catarrh, epidemic, Accounts of, — 335, 342, 354

Catgut, its Use in Obstructions of the Urethra, 378

Cavanilles, A. J. Dissert. Botanicæ, — 430

Cawley, Dr. Tho. singular Case of Diabetes, 286

Charles, R. on Consumptions, — 208

Chaussier, M. sur une Accusation d'Infanticide, 96

——————, Experiments relative to the Rupture of the Heart, — — 173

Cid, Don F. X. Tarantismo in Espana, — 97

Clarke, John, on an epidemic Disease of Lying-in Women, — — 417

——, Dr. Jos. on the Registry of the Lying-in Hospital, Dublin, — — 179

Colliander, Zach. de Moræa, — 419

Concanen, Tho. de Phthisi pulmonali scrophulosa, 421

Coste, Dr. J. F. Experiments with Opium in Lues Venerea, — — 7

Crawford, Dr. A. on animal Heat, — 207

Crichton, Dr. A. on the Astragalus Exscapus, 405

Crumpe, Sam. de Vitiis humorum, — 421

Cullen, G. prime Linee di pratica Medica, — 330

D. Page

Dahl, And. de Syftem. Veget. Linnei, — 212

Diabetes, fingular Cafe of, — 286

———, Experiments relative to, — 290

———, different Theories of, — 294

Dickfon, Dr. S. on Pemphigus, — 309

Difpenfatorium Fuldenfe, — — 421

Donovan, J. on venereal Diforders, — 210

Dryander, Jonas, on the Benjamin Tree of Sumatra, 80

Dublin Lying-in Hofpital, Abftract of its Regiftry, 199

Duffaufoy, M. fur la Gangrene des Hopitaux, — 97

E.

Edinburgh, Tranfactions of the Royal Society at, 88

Eichhorn, G. W. de Morbis gaftricis, — — 433

Electricity, Efficacy of, in a Cafe of Amaurofis, 389

Elfner, C. F. ueber die Inoculation, — 215

Evers, O. J. Bemerkungen und Erfahrungen, *ibid.*

F.

Fabbroni, J. Account of an Experiment in Hydrophobia, — — 69

Faye, M. fur les Eaux de Bourbon, — 101

Feller, C. G. vaforum Lymphaticorum Defcriptio, 96

Fifcher, J. L. de oeftro ovino, — — 432

Fiftula in Perinæo, Cafe of, — 378

Food, common, concife Obfervations on, — 86

Foot, J. on the Bite of a mad Dog, — 210

Ford, Edward, on Aneurifms, — 142

Forfter, J. R. Enchiridion Hift. Nat. — 432

Fofter, William, the experienced Farrier, — 327

Fourcroy, M. de, Elements of Chemiftry, — 326

———, fur l'Eau d'Enghien, — 429

Frederick II. King of Pruffia, Account of his laft Illnefs, — — 329

Frefnoy, M. du, fur le Rhus radicans, — 427

Fuchs, G. F. Chemical Text Book, — 428

G.

Gallini, S. Oratio Inauguralis, — 214

Gallot, M. fur une Fievre épidemique, — 218

Gehler, J. C. de ufu Cinnamomi in partu, — 420

Gentil, M. fur le Café, — — 103

Gevalin, J. E. de Phrenitide, — 95

Girdleftone,

Page
Girdleftone, Dr. Tho. on Hepatitis, —— 85
——————, de Hepatitide, —— 211
Girtanner, Dr. C. on the Venereal Difeafe, — 427
Gmelin, E. von Thierifchen Magnetifmus, — 216
Goodwyn, Dr. E. Connexion of Life with Refpiration, 325
Gouan, M. fur le Syfteme botanique de Linné, 103
Graves, Robertus, de Strabifmo, —— 422
Gren, F. A. C. de Aere fixo, —— 95
Guenet, M. Eloge de M. Bouvart, —— 96

H.

Haller, Alb. von, Bibliotheca Medicinæ Practicæ, 327

Hallmann, J. G. de Moxæ atque Ignis ufu, — 431

Hamerfley, Gul. de Rachitide, —— 91

Halloran, Gul. S. O', de Phthifi pulmonali fcrofulofa, 422

Hamilton, Dr. R. Duties of a Regimental Surgeon, 325

Handy, Gul. de Nutrimine Fœtus humani, - 422

Harwood, B. Synopfis of his Lectures on Anatomy, 86

Haft, H. R. de Caryophyllis, — — 430

Haygarth, Dr. J. ueber den Blattern, — 100

Healde, Dr. Tho. Tranflation of the new Pharm. Londin. 88

Heart, Obfervations on Ruptures of the, — 156

Heath, Joannes, de Afthmate, —— 91

Herrenfchwand, J. F. Traité des Maladies, — 424

Hippocrates, his Prognoftics, Tranflation of, — 85

Home, Everard, on the Properties of Pus, — 206

Hope, T. C. de Plantarum vita, —— 92

Hunter, Dr. John, on the Difeafes of Jamaica, 207

———, John, on the Effect of extirpating one Ovarium, 71

——————, German Tranflation of his Treatife on the Venereal Difeafe, — — 100

Hydrocele, fingular Cafe of, —— 109

Hydrocephalus internus, Hints relative to, — 122

Hydrophobia, Experiment relative to, —— 69

——————, fpontaneous, Cafes of, — 256, 267

J.

Jackfon, W. on Diftortions of the Limbs, — 90

Jahn, Frid. de Utero reverfo, ——— 95

James, Pinkfton, de Ictero, —— 92

Iberti, M. Obfervations fur les Hopitaux, — 424

Idees fur les Secours a donner aux pauvres Malades, 97

Jenner, J. C. on the Efficacy of Arfenic in Intermittents, 48

Inglis, And. de Rheumatifmo, —— 92

3 I 2 Infect

Page

Infect found in the Itch, Account of, —— 28

Johnstone, Dr. J. ueber den Nervenknoten, — 215

Jones, Philip, on Crookedness, —— 211

Joyand, M. Precis du Siecle de Paracelse, — 423

Ipecacoanha, Species of, at Rio Janeiro, — 69

————, said to operate with Severity, — 360

Itch, Insect found in the, described, — 28

——, two Species of, distinguished by the Ancients, 38

Juville, M. des Bandages herniaires, —— 37

Jurisprudence, medical, Elements of, —— 209

K.

Keate, T. Cases of Hydrocele, —— 210

Kelso, Dr. Hamilton, on elementary Air, — 86

Kissam, Ric. S. de Rheumatismo, —— 92

Koelle, J. L. de Aconito, — — 432

Koerber, J. F. de Nausea gravidarum, — 102

Kries, C. de Malignitate febrium, —— 433

Krzowitz, Trnka de, Historia Rachitidis, — 212

—————— Tympanitidis, - *ibid.*

L.

Lafosse, J. F. sur les Maladies de St. Domingue, 96

Landis, C. de Melancholia, —— 433

Lauth, Tho. Nosologia Chirurgica, — 213

Lavaud, M. de, Avis au Peuple, —— 214

Leake, Dr. John, Specimen Artis obstet. — 85

Leg, artificial, Account of one ingeniously contrived, 254

Lichens, Memoires sur leur Utilité en Medecine, 425

Lieb, J. W. ueber die Eispflanze, —— 99

Loftie, W. Case of Exfoliation of Part of the upper Jaw, —— —— 57

Low, Jac. de Hæmorrhoide, —— 92

Lowndes, F. on medical Electricity, —— 87

Lucas, James, on elastic Bandages, —— 44

———— Amputation, —— 223

Ludwig, C. F. Historiæ Anatomiæ brevis Expositio, 95

Lundmark, P. de Restione, —— 430

Luxmore, Henr. de Scorbuto, —— 92

M.

Macartney, J. de Apoplexia, —— 92

Mac Causland, Rob. de Hepatitide, —— *ibid.*

Mackintosh, Jac. de Actione musculari, — *ibid.*

Manchester,

Page

Manchester, German Translation of the Memoirs of the Society at, —— —— 429

Marsonnat, M. de, sur les Eaux de Charbonniere, 214

Martyn, Tho. Botanical Plates, — 326

May, Dr. William, on the Treatment of Phthisis, 268

——————, his Dissertation de Typho, 327

——————, his Account of an epidemic Catarrh, 342

Maxwell, Gul. Exp cum diversis Aeris speciebus, 93

Mehlis, G. H. de Excitantium usu in Febribus, 101

Mesembryanthemum crystallinum, its medicinal Properties, —— —— 100

Metherie, M. de la, sur les differentes Especes d'air, 329

Michels, F. ueber die Mineral Wasser der Reichstadt Achen, —— —— 104

Midwifery, periodical Work relative to, in Italian, 330

Moffatt, Dr. J. his Translation of the Prognostics of Hippocrates, —— —— 85

Monro, Dr. Alex. on the Bursæ Mucosæ, — 207

——, Dr. Donald, on Medical and Pharmaceutical Chemistry, —— —— 211

Morison, Fr. de Dyspepsia, — — 93

Moseley, Dr. B. on Tropical Diseases, — 89

Moufet, Tho. his Description of the Itch Insect, 29

Moultrie, Jac. de Actione et usu Emeticorum, 422

Murray, J. R. de Abortu, — — 93

N

Neale, Mr. on Nervous Complaints, — 418

Nessi, G. Instituzione de Chirurgia, — 99

Nisbet, Dr. W. on the Venereal Disease, — 85

Nolde, A. F. circa sexus differentiam, — 433

O.

Opium, Experiments with, in Lues Venerea, — 7
Orlandi, P. de Variol. Inocul. — 433
Ovarium, Extirpation of one, Effects of, — 71

P.

Panzani, Jac. Malattia Verminosa della Vescica, 217

Paris, Medical Police at, Memoirs relative to, 103

Partington, Miles, Case of Amaurosis, — 389

Peake, J. Review of J. Foot's Observations, — 90

Pearson, Dr. G. of Phosphorated Soda, —— 393

——, John, Principles of Surgery, — 88

Peart, Dr. E. on Animal Heat, —— 208

Pemphi-

Page

Pemphigus, Obfervations relative to, — 309

Perinæum, Cafe of Tumor in, after Delivery, 119

Pharmacopeia, Coll. Med. Londin. — 88

————————, Englifh Tranflation of, *ib.*

———————— Obfervations on, 87, 210

Phillip, Governor, his Account of Tipioca, — 69

Phthifis, Remarks on the Treatment of, — 268

Phyfic, Compendium of the Practice of, 429

Pilhes, M. fur les eaux d'Ax et d'Uffat, — 101

Pifo, G. his Account of Tipioca, — 68

Planer, J. J. Index plantarum Erfurt. — 211

Portal, M. fur les Noyés, la Rage, &c. — 102

————, on Ruptures of the Heart, — 156

Potton, And. de Colica, — 93

Principia Botanica, — — 84

Q.

Quillin, Gul. de Ictero, — 422

R.

Radloff, F. V. de Myriftica, — 431

Raffler, H. J. on Inoculation, 429

Reeve, Tho. Cafe of Tumor, *in Perinæo*, after Delivery, 119

Reform, Medical, Plan of, — 417

Relhan, R. Floræ Cantab. Supplementum alterum, 419

Rengger, Alb. de Conftit. ævi noftri febrilis, — 213

Retzius, A. J. de Vermibus Inteftinalibus, — 95

Reynier, M. fur l'Hift. nat. de la Suiffe, — 429

Reynolds, Jac. de Calculo Urinario. — 93

Rhades, F. H. de Temperamentis, — 95

Rhus Radicans, Account of its Properties, — 427

Riboli, Aug. full 'ufo del fuoci, — 99

Richter, A. G. Traité des hernies, — 425

Rigby, Edw. Chemical Obfervations on Sugar, 99

Riollay, F. Introduction to the Study of Fevers, 417

Robertfon, Jac. de foetus humani Nutrimento, 422

Roth, A. G. Flora Germanica, — 432

Rowley, Dr. W. on the ulcerated fore Throat, 209

Roy, A. le, Hift. Natur. de la Groffeffe, — 218

Ruffel, James, Cafe of Hydrophobia, — 256

Ryan, Dr. M. on Pulmonary Confumption, — 208

S.

Sachlen, J. F. de ufu Inuftionum vario, — 420

St. John, Dr. J. Chemical Nomenclature, — 90

Sayani,

Page

Savani, G. M. sulla Materia Zuccherina, — 98

Scarpa, Anton. Annotationes Academicæ — 419

Schaeffer, C. F. G. de abusu Clysmatum, — 403

Schæffer, G. B. de Angina Pectoris, — 132

Schæffers, J. C. G. Med. Ortbeschreibung der Stadt Regensburg, — — 329

Scheele, C. G. Opuscula Chemica, — 438

Schlereth, F. A. Dispensatorium Fuldense, — 421

Schlickum, E. de Sympathia, — 429

Schmelzer, F. A. de Probabilitate vitæ, — 212

Schmid, G. A. R. de Sale Ammoniaco, — 433

Schoepf, J. D. Materia Medica Americana, — 95

Schregerus, B. G. Pelvis Brutorum cum Humana Compar. 420

Schwartze, J. F. Observationes Medicæ, — *ib.*

Scuderi, F. M. dell 'Estinzione del Vajuolo, — 217

Selle, C. G. Krankheitsgeschichte des Friederich's II. 329

Seton, Patr. B. de Paralysi. — 93

Short, Jac. de testium tumore Gonorhœæ superveniente, 422

Siebold, C. C. Hist. tumoris alveolaris, — 431

Siles, Don B. P. y. de una nueva Especie de Corea, 98

Simmons, Dr. S. F. of the Epidemic Catarrh, 335

Skeete, Dr. T. German Transl. of his Work on the Bark, 429

Smith, Aug. de Morbillis, — 93

——— Franciscus, de Inflammatione Pneumonica, 423

———, Joannes, de Ophthalmia, — *ib.*

———, Dr. S. S. on the Variety of Complexions in the Human Species, — — 415

Smithwick, R. de Erysipelate, — 94

Smyth, Dr. J. C. his Edition of Dr. Stark's Works, 89

Soda, phosphorated, Account of, — 393

Sparrow, Richard, Cases by, — 109

Stanistreet, H. de Gonnorhœa, — 94

Stark, Dr. W. Works of, — 89

Steidele, R. on the Management of lying-in Women, 428

Stevenson, James, Case of fatal Suppression of Urine, 382

Stolte, C. H. de Vitriolo Albo, — 101

Stomach, Case of a fatal Inflammation of, — 382

Straehl, J. F. de bilis Natura, — 102

Straith, Alex. de Amenorrhœa, — 94

Struve, M. sur l'Hist. nat. de la Suisse, — 429

Sudor, Anglicus, its Resemblance to Epidemic Catarrh, 373

Taplin

T. Page

Taplin, W. Syftem of Farriery, — 326

Tarantula, Publications relative to, — 97, 98

Tartar, Emetic, Effects of a large Dofe of, — 61

Taylor, Joannes, de hæmorrhagiis, — 94

Thompfon, Sir Benj. Experiments by — 201

Tipioca, Account of, — 67

Titus, S. C. de Acido Vegetablium, — 431

Tudefq, M. fur l'Inoculation, — 214

Turner, T. Cafe of Amputation in the Middle of the Foot, 54

V.

Vachier, M. Methode pour Traiter toutes les Maladies, 423

Valentini, Onof. Difcorfo Medico-Chirurg. — 93

Venereal Difeafe, Experiments with Opium in, 7

———, Effects of the Aftragalus Exfcapus in, 405

Underwood, Dr. M. Surgical Tracts, — 209

Vogler, J. P. Pharmaca Selecta, — 429

Urine, Cafe of a fatal Suppreffion of, caufed by Poifon, 382

Ufteri, P. Bibliotheca Magnetifmi Animalis, — 213

W.

Wallace, Tho. de Angina Tonfillari, — 94

Walter, Tho. Flora Caroliniana, — 419

Warren, Dr. J. on Hydrocephalus Internus, — 122

Wafferberg, F. A. von, Chem. Abhand. vom Schwefel, 216

Watfon, Jac. de Amenorrhœa, — 423

Watfon, Dr. Richard (Bifhop of Landaff) Chemical Effays, 87

Werner, P. C. F. Vaforum lymphat. Defcriptio, 96

Weftrumb, J. F. Phyfico-Chemical Effays, — 216

Wichmann, J. F. on the Infect found in the Itch, 28

Wilkinfon, Geo. Cafe of Fiftula in Perinaeo, 378

Willdenow, C. L. Floræ Berolin. Prodromus, 420

Wilmer, B. on Herniæ, — — 90

Wilfon, Jof. Nic. de Tetano, — 423

Wrifberg, H. A. de Uteri Refectione, — 94

Z.

Zeigler, C. J. Medical Obfervations, — 100

Zorn, L. Mifcellaneous Obfervations, — 329

